# Testing a faith-placed education intervention for bowel cancer screening in Muslim communities using a two-group non-randomised mixed-methods approach: Feasibility study protocol

**DOI:** 10.1371/journal.pone.0293339

**Published:** 2024-03-15

**Authors:** Marimba Carr, Claire Thompson, Tara Berger-Gillam, Joanne Freedman, Nigel Smeeton, Salman Waqar, Daksha Trivedi

**Affiliations:** 1 Screening and Immunisations Team, NHS England and Improvement, East of England, Welwyn Garden City, Hertfordshire, United Kingdom; 2 Centre for Research in Public Health and Community Care, University of Hertfordshire, Hatfield, Hertfordshire, United Kingdom; 3 Norwich Medical School, University of East Anglia, Norwich, United Kingdom; 4 Nuffield Department of Primary Care Health Sciences, University of Oxford, Oxfordshire, United Kingdom; Freelance Consultant, Myanmar, MYANMAR

## Abstract

**Background:**

Inequalities exist in uptake of bowel cancer screening in England with low uptake in areas with high deprivation and amongst certain ethnic and religious groups. Individuals from these groups are more likely to receive a late diagnosis of bowel cancer. Uptake in Muslim communities, for example, has been shown to be lower than in the general population. Culturally adapted interventions are needed to address these inequalities. This feasibility study aims to assess the acceptability and accessibility of an educational faith-placed bowel cancer screening intervention in the East of England, alongside its impact on bowel screening uptake. It was developed by the British Islamic Medical Association in partnership with community stakeholders and professionals.

**Methods:**

Ethical approval was granted on the 27 October 2021, REC reference number 21/EE/0231. A two-group non-randomised feasibility mixed methods study will be conducted, using surveys, focus groups and semi-structured interviews. Participants eligible for bowel screening will be recruited through local mosques and community venues. We aim to recruit 100 participants to the intervention group and 150 to the comparison group (not receiving the intervention). Intervention group participants will complete a survey at baseline, post-intervention and at six-month follow up. Comparison group participants will complete a survey at baseline and at six-month follow up. Outcomes will include: intention to take up screening; actual screening uptake; knowledge, attitudes, barriers and facilitators towards screening. Regional screening hub records will be used to ascertain actual screening uptake at six-month follow-up. Quantitative survey data will be summarised using descriptive statistics (e.g., proportion), and exploratory univariate analysis will be undertaken (e.g., chi-squared test). Two focus group interviews will be conducted with intervention group participants (with up to 16 participants). Semi-structured interviews will be conducted with 10 clinicians delivering the intervention to explore the acceptability of the intervention, training, and delivery. All qualitative data will be subject to a general inductive analysis.

**Discussion:**

The findings will inform how faith-placed interventions can be implemented to increase uptake of bowel cancer screening, and potentially other health promotion programmes, to address health inequalities in ethnically diverse communities in England.

## Introduction

### Background

Screening can save lives and improve quality of life through early identification of a condition. It can also reduce the chance of developing a serious condition or its complications [1]. In England, for over 450,000 people a year, screening produces results that lead to further tests or treatments, with cancer screening programmes preventing 9,000 deaths per annum [2]. Inequitable access to healthcare and preventive services such as screening can lead to significant, avoidable disparities in health outcomes for some groups in society. Ethnicity is not well recorded in screening data, however studies and surveys have shown lower uptake of screening amongst ethnically diverse groups [3–7]. Screening services also consistently underserve low-income groups, who carry a disproportionate cancer burden in England [8–10].

Bowel cancer screening aims to identify cancer early, allowing treatment to be given before it can progress, and improve health and survival outcomes. More than 9 in 10 people survive bowel cancer for five years or more when diagnosed at an earlier stage, compared to 1 in 10 when diagnosed at a late stage [11].

On average around 64% of people took up the offer of bowel cancer screening in England in 2019/20 [12]. However, take-up is much lower in some groups including those in areas of high deprivation and in ethnically diverse communities. There are important variations in the uptake of bowel cancer screening by ethnic groups and religion, with lower uptake in Muslim and South Asian groups, s as compared to those who identified as White and Christian [13]. Emotional barriers such as fear, embarrassment and anticipated shame, as well as low perceived risk, might contribute to explaining lower screening coverage for some ethnic minority communities, such as low uptake of cervical cancer screening amongst ethnic minority women [14]. In addition, people from ethnically diverse backgrounds are more likely to receive a late diagnosis of bowel cancer [15].

The disparities identified in a Health Equity Audit conducted in 2020 by Public Health England, East of England (unpublished) were consistent with these statistics. For example, the audit found that for GP practices in the East of England, every 1% increase in deprivation (indicated by the Income Deprivation Affecting Older People Index) was associated with a 0.79% decrease in bowel screening uptake. Every 1% increase in non-white ethnicity was associated with a 0.16% decrease in bowel screening uptake. It also highlighted that men and people with disabilities were also less likely to access bowel cancer screening.

The East of England includes localities that have both a high proportion of Muslim residents [16] and low uptake of bowel cancer screening and areas with high levels of deprivation [17]. Culturally adapted interventions within faith settings may improve participation in cancer screening and help to address health inequalities [18–22]. A better understanding of both screening uptake and screening outcomes, analysed by ethnicity and by religious groups (acknowledge that there is likely to be overlap) has the potential to inform more targeted education and informed choice strategies [13].

Culturally sensitive interventions are needed to inform communities with low screening uptake about the need for screening and to overcome barriers such as disinformation, religious objections and cancer fatalism [13, 23, 24]. The British Islamic Medical Association [BIMA] screening intervention, which received a 2019 Royal Society for Public Health: PHE commendation [25], was designed to address this need.

Public Health England, alongside community stakeholders, identified Luton and Peterborough (East of England, UK) as areas that could benefit from the intervention based on bowel cancer screening uptake, levels of deprivation, and ethnic diversity of the populations. The proposed study will determine the feasibility of the intervention.

### The intervention

The BIMA cancer screening intervention uses an adapted presentation slide deck from Cancer Research UK, a national cancer charity. These slides are very lightly modified. This includes having a single slide to motivate attendees through the Islamic injunction to uphold good health. It includes graphics that are tailored to Muslim culture (i.e. women wearing hijab). A slide with publicly available local data that contextualises the shortfall in cancer diagnosis and survival in the mosque community has also been added. The rest of the slides are unaltered and provide a one-hour group education session on bowel cancer screening. This covers screening benefits and harms, how to complete the test, and is followed by a question-and-answer session.

The planned intervention outlined in this protocol will be delivered by local, trusted, senior clinicians who are known to the community. The benefit of this approach will be considered as part of the study.

This innovative conceptual framework positions the intervention as ‘faith-placed’, rather than ‘faith-based’. While there are benefits to ‘faith-based’ interventions there are also risks and ethical considerations [23]. Faith-placed interventions aim to use faith settings such as mosques to target specific communities, without co-opting religious and health messaging [26]. Given the importance of family units in some Muslim cultures, Muslims may value family or community-based approaches to health improvement [27]. This study will therefore also aim to include relevant family and community members. As mixed-gendered groups may limit participation from these communities, we will have gender concordant groups, with male and female groups to be delivered by male and female clinicians respectively. Face-to-face delivery in the community (for example at the mosques after Friday prayers) will be the preferred mode of engagement.

However, as an alternative method of delivery, participants will be offered the option to access the intervention virtually due to the current COVID-19 pandemic restrictions (at the time of planning the feasibility study). This will be offered as a recorded information session with a platform for asking questions or as a live online meeting (via Zoom or Microsoft Teams) and will also serve to include groups which may have an affinity to a faith sensitive intervention but have access issues to the mosque space.

There is already tentative evidence suggesting the potential effectiveness of this approach. A cancer screening promotion event was delivered across 39 faith venues for the Muslim community in 2019. A survey was conducted before and immediately after the event (on the same day). A small number of those who attended the event completed the surveys, (166 of the 900, 18%). Within this group, self-reported intention to take up screening increased from 38% prior to receiving the information to 90% after the event [28]. Further and comprehensive evaluation is now required to establish the acceptability and effectiveness of the intervention to inform approaches to further roll-out.

The findings of this feasibility study will be of regional and national relevance to the implementation and uptake of bowel screening in culturally diverse groups. It is anticipated that if the approach is successful in establishing the feasibility, acceptability, and an increase in screening uptake, the next step would be wider trial rollout in other areas in England where the same issues of deprivation and health inequalities apply within the region.

## Aim and objectives

### Aim

To carry out a feasibility study to assess the acceptability and accessibility of an educational faith-placed bowel cancer screening intervention in the East of England, alongside its impact on bowel screening uptake.

### Objectives

- Assess knowledge, attitudes, and behaviours to bowel cancer screening at baseline, post-intervention (intervention group only) and at six-month follow-up
- Assess intention to undertake screening, as well as validated screening uptake:
  - Self-reported intention to undertake bowel cancer screening
  - Number/ proportion of participants requesting a bowel cancer screening kit
  - Number/ proportion of participants returning a bowel cancer screening kit
- Understand the feasibility, accessibility, and acceptability of the intervention for participants and for health professionals delivering the intervention
- Identify barriers and facilitators that might impact uptake of bowel cancer screening

## Methods

The study was granted ethical approval on the 27 October 2021, REC reference number 21/EE/0231. The study flow chart is available in S1 File.

## Study design

This study will be a two group, mixed methods non-randomised feasibility study. S1 Fig below summarises the study design.

### Study components

The intervention will be evaluated through a mixed-methods approach in line with the Medical Research Council framework for process evaluations [29]. There will be two main elements to the evaluation:

Phase 1: Outcome evaluation

1. **Participant surveys** completed by those receiving the intervention and by those in the comparison group (not receiving the intervention)

Phase 2: Process evaluation

2. **Focus groups** with participants receiving the intervention
3. **Semi-structured interviews** with health professionals delivering the intervention

#### Outcome evaluation

In phase one, intention to take up screening will be assessed through a self-reported survey completed at baseline, a post-intervention survey completed immediately after, or within 5 days of, receiving the intervention (intervention group only), and at six-month follow up (intervention and comparison group). A six-month interval was chosen to give the participants sufficient time to request and complete the test while keeping the attrition associated with longer follow-up to a minimum. A larger number of comparison group participants is planned as, based on the delivery of the intervention, recruitment to the comparison group is likely to take less resource than for the intervention group.

At six-month follow up, participants in both groups will be asked if they have requested a faecal immunochemical test (FIT) screening kit, and if they have completed and returned the test (if eligible for screening during the study period).

The Eastern Bowel Cancer Screening Hub will be asked to run a report at six-month follow up to identify participants in study and comparison groups who have requested and/or returned a kit. This will validate participant self-reported data.

#### Process evaluation

In phase two, participants will be invited to attend a focus group to assess the feasibility and acceptability of the intervention. Two focus groups will be conducted with a maximum of 16 people who have received the intervention (6–8 in each focus group). These will take place post-intervention.

Semi-structured interviews with clinicians and other health professionals delivering the intervention (10 clinicians) will be carried out to determine the feasibility and acceptability of the intervention from their perspective. This will include the process of recruiting and training clinicians to deliver the intervention with their communities.

## Participants and sample size

This study will be conducted with four groups of participants:

- Individuals and/or their family members/carers identified through mosques/community groups **receiving the intervention**
- Individuals and/or their family members/carers identified through mosques/community groups **not** **receiving the intervention** as a comparison group
- Individuals who attend the intervention session, and provide consent to be contacted to be invited to participate in **focus groups**
- Clinicians/other health professionals recruited to deliver the intervention participating in **semi-structured interviews**

### Inclusion and exclusion criteria

#### Intervention and comparison groups

Inclusion criteria.

- Individuals aged 56 years and over (in line with the National Bowel Cancer Screening Programme eligibility, 56 was the minimum age for inclusion in the screening programme at the time of protocol development) [30]
- Individuals aged under 56 years participating on behalf of a friend/relative/someone they provide care for who is aged 56 years and over. The justification for this is that a member of the Mosque or Muslim community may be living with or caring for someone who is eligible for bowel cancer screening, and so can attend on their behalf. The surveys will capture whether they are responding on behalf of someone else
- Use of English as a first language
- Use of another language (including Bengali and Urdu) if translation support is available

Participants who require support with translation will be encouraged to ask family members where possible; alternatively, community volunteers will also be available to support participants with the completion of the relevant surveys.

*Exclusion criteria*.

- Individuals with a recent bowel cancer diagnosis and/or currently undergoing treatment
- Aged under 56 years old (if not representing an individual who meets the eligibility criteria)
- Use of a language for which support from family members and/or community volunteers cannot be found

Feasibility studies do not include formal sample size calculation [31]. As a feasibility study, a sample of 250 participants will be obtained consisting of 100 in the intervention group and 150 in the comparison group. As well as being a pragmatic sample size, this will provide a 95% confidence interval for the proportion of participants in the intervention group who request and/or complete and return a bowel screening kit to an accuracy of ±0.1 and for the participants in the comparison group to an accuracy of ±0.08. Recruitment rates and baseline to 6-month follow-up percentage attrition will be estimated for the two groups. The effects sizes for the differences in proportions between the two groups will give an estimate of the sample sizes required in a future definitive study.

All participants attending the intervention session will be asked if they would be willing to participate in a focus group. We will conduct four focus groups, with 6–8 participants in each (total of 32 participants, at most). We will also conduct semi-structured interviews with each of the clinicians involved in the delivery of the intervention. It is anticipated that this will be with 10 individuals.

### Sampling approach and recruitment

The population for the study will be individuals attending mosques and Muslim community groups in Luton and Peterborough. We will target mosques that are in catchment areas of GP practices with low bowel cancer screening uptake. The process for identifying potential mosques to participate in the study is outlined in S1 Appendix. Discussions with community leaders will take place to identify which mosques would be best placed to deliver the intervention, and which mosques would be best to support recruitment of participants for the comparison group. Whilst there are limitations to this, and the potential to introduce bias, the intervention is based on the model that the clinicians deliver the intervention within their communities. As a feasibility study, it will also be helpful to work with the mosques and communities who are willing to engage.

Gender specific sessions will be planned in consultation with community leaders, clinicians and patient public involvement representatives to ensure that both men and women are able to participate in the study. As a minimum in both Luton and Peterborough, we plan to work with one mosque for men and one community group for the women in the intervention group plus two mosques/two community groups for the comparison group.

Representatives of those who are eligible for the study are being recruited to ensure that those who rely on family support can participate. Some of those who are eligible may prefer to ask a representative to attend the session rather than attend themselves, due to language or other barriers.

Strategies and approaches to promote the study and to support recruitment of participants for the intervention and comparison groups, will be agreed in consultation with the project advisory group and the patient public involvement (PPI) representatives. These are likely to include 1) information provided at prayer sessions by the Imam (or community group meetings by the group leader); 2) using social media to share links to written information as well as short, pre-recorded video clips in appropriate languages, 3) local radio, 4) printed material in relevant languages, and 5) case studies. We will also engage with key stakeholders e.g. local pharmacists, schools (e.g. family workers), local councillors, women’s groups, and GP practices to raise awareness of the study.

Clinicians to deliver the intervention will be recruited through the BIMA volunteer network which has over 6,000 members who are professionally vetted and are trained to deliver the presentation and anticipate questions. These professionals are themselves part of the target communities. Individual participants will be recruited through Muslim community/faith groups in the target areas identified, using purposive and opportunistic sampling and recruitment methods, with community leaders and clinicians within the communities promoting the sessions.

Peer-researchers with competence in at least two of the relevant languages (Urdu and Bengali) identified through the mosques will be trained to support the completion of consent forms and study surveys. They will also be trained to support the facilitation of the focus groups. We anticipate that this will avoid the potential situation of lack of trust or openness that may arise if non-Muslim researchers are leading the focus group discussions. Once identified, the research team will hold a virtual training session with the peer-researchers. The training will include an overview of the study, the role of the peer-researchers, with a particular focus on the completion of the consent form and the baseline and post intervention surveys, how to conduct the focus group. Anticipated challenges, and how to overcome them, will also be discussed.

Given the exploratory nature of this study, it was thought that a non-randomised approach would be operationally more practical. Sites will be identified opportunistically, which did not lend itself to randomised design. As this is not a randomised study, participants will be able to choose to be part of the comparison group if they attend a venue which is acting as an intervention site and prefer not to access the intervention, and participants who attend a venue which is acting as a comparison group site will be able to complete the intervention if they wish to receive the intervention. Data analysis will be carried out on individual responses. Given the planned sample size it will be difficult to investigate outcomes by ethnic group within the Muslim population.

To encourage participation in both the intervention and the study groups in this study, we propose to make a monetary donation of five hundred pounds to each participating mosque. Given the limited study budget it will not be possible to provide individual incentives.

One intervention session will be delivered at each intervention site as part of the feasibility study. Part of the feasibility study will be to assess how many participants attend the session as a result of the promotion as outlined above.

## Evaluation data collection and protocol

### Phase 1: Outcome evaluation

#### Participant surveys

Participants in the intervention and comparison groups will provide data through self-completed surveys. The survey tools included as supporting information in S2–S4 Appendices. Participant information sheets will be translated into Bengali and Urdu and will be provided to participants in the intervention and comparison groups ahead of asking them to participate. Printed copies will be made available at the participating mosques. These will be available in digital and hard copy.

Where possible, participants will be asked to complete the consent and survey forms digitally, shared via text message or email. This is to reduce the administrative burden of data entry given the limited study resources. A digital consent form will be included at the start of the baseline survey to gain participant consent. Feedback from stakeholders suggested that we host ‘drop-in’ sessions where peer-researchers are available to support participants with survey completion. We will explore this and plan accordingly with the participating venues for each of the three surveys.

Participants in the intervention and comparison groups will be asked to complete a baseline survey. A survey used in a previous study [32] has been adapted to suit the current study. Participants receiving the intervention will need to complete this before attending the information session (either face-to-face or virtually). For those attending the information sessions face-to-face, and who have not yet completed the consent and baseline survey online, paper forms will be available. Peer-researchers will be available to support participants to complete the survey. If individuals do not fill in the baseline survey, they will not be included as participants, but they will still be permitted to attend the information sessions.

Participants in the control group will be provided with the link to the online consent and baseline survey forms. This information will be available at the participating control sites for participants to request, or they will be able to sign up to participate and the link will be sent to them by text message or email. ‘Drop in’ sessions to support with survey completion at control sites will also be explored.

Intervention participants will be asked to complete a post-intervention survey ideally immediately after receiving the intervention (digitally, or paper form), otherwise within 5 days of receiving the intervention. Those attending face-to-face will have the option of completing a paper form, and if this is done immediately after the session, peer-researchers will be available to support if they need it.

Participants in both groups will be asked to complete a self-reported follow-up survey at six months, when a link to the electronic survey will be shared via email or text message. We will work with the participating mosques to share reminders with the aim of increasing the number of responses. A reminder email and text will be sent if no response is received within one week of the required deadline. The ‘drop in’ offer will also be considered.

Primary outcomes measures will be the acceptability and accessibility of the intervention for participant as well as clinicians delivering the intervention. These will be assessed via the focus groups and semi-structured interviews respectively.

Secondary outcome measures will be intention to screen and use of screening as reported by the participant. Intention to screen will be assessed by the likelihood of participation in bowel screening when invited and the likelihood of requesting a screening kit, if eligible to receive one, measured at baseline, post-intervention, and six months. Use of screening will be measured by the request of a faecal immunochemical screening kit and completion / returning of the test, if eligible for screening, as reported by the participant at six months.

#### Data validation

Participants will be asked to provide name, address, date of birth and NHS number (if known). This will enable us to validate actual screening intention/uptake through the Screening Hub records (kits requested/returned) between participants in the intervention and the comparison groups. We will carry out a validation at six months post-intervention.

### Phase 2: Process evaluation

#### Focus groups

All data collection for this phase will be conducted post-intervention and with the intervention group only. Two focus groups will be conducted with participants who have received the intervention. Each focus group will have 6–8 participants (up to 16 in total). Peer-researchers will support the delivery of the focus groups. The focus group discussion will examine experiences of the intervention, accessibility, acceptability and attitudes towards screening. The research team are from diverse ethnic backgrounds, including some Muslim researchers, and we will endeavour to be sensitive to the preferences and dynamics of participant groups when conducting fieldwork. Focus group participants will be compensated for their travel and subsistence costs. The focus groups will be single-sex [33]. We will take a gatekeeper-led and emergent approach to this. Gatekeepers will be the leaders and volunteers at the mosques who are typically responsible for organising community events and are well known and trusted members of their local communities.

#### Semi-structured interviews

We will conduct semi-structured interviews with each of the clinicians involved in the delivery of the intervention. It is anticipated that this will be with 10 individuals. The interviews will most likely be conducted remotely (although in person where possible) and will explore how the professionals became involved in the intervention, the training they received, the experiences and perceptions of delivery, and suggestions for improvements and refinements. These interviews will be one-to-one (although in pairs if requested by participants) and will last around 40 minutes.

A summary of the data management plan can be found in S2 Fig.

## Data analysis plan

### Phase 1: Outcome evaluation

Analysis will be undertaken comparing responses between the intervention group and the comparison group using SPSS. As the primary focus in a feasibility study is on the methods for dealing with the feasibility objectives rather than statistical testing [31] results will be presented using descriptive statistics (e.g., mean, proportion) and associated 95% confidence intervals. In addition, to gain further insight into an appropriate sample size for a definitive study, exploratory univariate statistical analyses (e.g., t-tests, chi-squared test) will be performed. At baseline we will compare demographic data and eligibility for bowel screening to determine differences in baseline characteristics between the intervention and comparison groups. This will inform our interpretation of any differences in outcomes and enable us to identify demographic and eligibility differences in those who chose to take up the intervention compared to those who did not. Likert Scale responses to questions assessing knowledge, attitudes and intention to take up screening will be gathered at baseline (both groups), post-intervention (intervention group only) and at 6-month follow-up (both groups). This will enable us to compare understanding of screening between groups at baseline and 6-month follow-up, and, for the intervention group, short-term changes in understanding following the intervention.

### Phase 2: Process evaluation

Focus groups will be digitally recorded and shared securely (by encrypted file) with the service commissioned to complete the transcription. Once transcribed, that data will be returned securely, and the recordings will be deleted by the transcription service. A data sharing agreement is in place between the University of Hertfordshire and the transcription service. Remote interviews will be recorded via Zoom or MS Teams. Recordings will be shared with the transcription service as for the focus group data. Data will be stored securely on University of Hertfordshire and NHS systems. Information will be shared via secure platforms or encrypted in line with information governance requirements.

Interviews and focus groups will be transcribed verbatim, uploaded to NVivo software and subject to a General Inductive Approach (GIA) analysis [34]. Fieldnotes will also be uploaded as part of the data set and subject entered into NVivo for analysis. The analytical aim will be to identify and explore links between contexts (C), mechanisms (M) and outcomes (O). Data will be coded into COM dyads and developed into a framework to generate mid-range theories around processes and context [35]. Particular attention will be paid to discordant voices. The research team will meet regularly to achieve consensus in data interpretation.

## Patient and public involvement

The project will take a collaborative approach, which values co-production with partners in the region. Clinicians from the British Islamic Medical Association (BIMA) who are also part of the Muslim community, and who are Muslim themselves, have been and will continue to be involved in designing, planning and delivering the project.

Members of the Public Involvement in Research Group (PIRG) at the University of Hertfordshire, who are also Muslims, commented on an initial project proposal. The PIRG will continue to be involved throughout and offer feedback and critical guidance on the project including ethics, recruitment and dissemination.

Community leaders will also be involved and consulted throughout the project to ensure that they meet the needs of their communities.

The project has been discussed at the NHS England Bowel Cancer Screening Inequalities Forum. It has also been discussed at a local Cancer Survivor Patient Forum to get their views.

There are two patient and public involvement representatives on the advisory group. They are consulted on different aspects of the project. Examples include 1] planned approaches to ensuring that women can participate in the project 2] use of appropriate language in participant information and consent forms and survey questions (not too technical, translated if possible).

## Ethics

### Approvals

University of Hertfordshire are the study sponsors and have reviewed and approved the study protocol and the ethics application, protocol number HSK/SF/NHS/02971.

Ethical approval was sought through the Integrated Research Application System ((IRAS)). The Health Research Authority ((HRA)) confirmed that full HRA approval was not required as it did not involve NHS sites. As the study does involve a sensitive topic and there is an element of burden to the participants, the study required a Research Ethics Committee review ((rather than a proportionate review)). The project team met with the Cambridge Central Research Ethics Committee ((REC)) on the 8 October 2021. The REC required minor changes to the protocol and the participant information sheet to include detail on how any safeguarding issues identified would be managed. The study documents were revised accordingly. Ethical approval was granted on the 27 October 2021, REC reference number 21/EE/0231.

### Participant information and consent

Participant information (translated into one or two additional languages) and a consent form will be available prior to participation in the study for those in the intervention and the comparison groups as well as the clinicians recruited to deliver the intervention. If required, we will also allow for verbal consent to be obtained at face-to-face intervention sessions (agreed in the ethical approval), as Patient and Public Involvement representatives indicate that participants may be reluctant to provide a signature or written consent.

Participants will be able to withdraw from the study at any time.

The UK Policy Framework for Health and Social Care Research will guide the approach to confidentiality aspects of this study [36].

Participants will be asked to provide patient identifiable information to validate objective data on actual screening uptake. Only researchers who require specific participant information will be given access.

Responses and participation will remain anonymous in any published materials relating to the study.

### Safeguarding and support

If information disclosed by participants (through surveys, the intervention session, or the focus groups) raises any safeguarding concerns, a member of the project team will contact the individual to discuss this with them, as outlined in the consent form. We will aim to get their consent to refer them to an appropriate individual or organisation who may be able to provide support. Depending on the nature of the concern, this may be the Imam, the clinician delivering the intervention session, or the individual’s GP. If more serious in nature, the safeguarding policies and procedures of the leading organisations (University of Hertfordshire and NHSE England and Improvement) will be followed. It may also be appropriate to refer to the safeguarding policies of the participating mosques/ community groups.

Individuals who have been affected by cancer, directly or indirectly, may find some of the questions in the survey, or information in the intervention session upsetting or difficult. Details of individuals and organisations who may be able to offer support have been included in the participant information sheet. This includes the clinician delivering the intervention session; the Imam or community leader in their role of providing pastoral support; or the individual’s GP. Details of cancer charities have also been included:

- Bowel Cancer UK: visit www.bowelcanceruk.org.uk
- Cancer Research UK: visit www.cancerresearchuk.org or call 0808 800 4040
- Macmillan Cancer Support: visit www.macmillan.org.uk or call 0808 808 0000

The surveys, participant information sheet and consent form have had input from patient and public involvement representatives to ensure that they are appropriate and culturally sensitive.

The clinicians delivering the intervention will also ensure that the information is delivered in a culturally sensitive way. Clinicians are trained and experienced in talking to patients about health and care issues.

## Discussion

### Value of the intervention

Evaluating this intervention is important because it will contribute to the evidence-base for culturally adapted interventions and inform adaptations. The current lack of evidence is a barrier to implementation and the sharing of good practice that could help address health inequalities.

If the research shows that the intervention increases screening uptake in those who receive the intervention, compared with those who do not, and participants find it acceptable and accessible, this will inform a wider roll-out of the intervention. This will result in more people benefiting from the intervention and the research.

### Potential usefulness of the findings

The results from this evaluation will also inform how faith-placed interventions can be implemented to increase uptake of bowel cancer screening, and potentially other types of cancer screening or health promotion programmes, contributing to the addressing of health inequalities in ethnically diverse communities in England.

### Anticipated difficulties

Difficulties anticipated with the study include the recruitment and retention of participants; complete and timely data collection, including consent; and challenges with intervention delivery due to COVID-19. We have developed approaches to address the anticipated challenges as discussed in the relevant sections in the protocol. These will all be assessed as part of the feasibility study.

## Current study status (as of November 2021)

This study has recently been granted ethical approval. The next steps are:

- Further stakeholder engagement including identifying intervention and comparison sites, working with local faith leaders and community groups
- Finalising the intervention resources and study tools
- Intervention delivery and evaluation to begin in December 2021

## Current study status (as of June 2023)

- Intervention sessions have been completed
- Focus groups are being conducted
- Follow up surveys are beginning to be collected
- Next steps include requesting data from the East of England Bowel Screening Hub on actual screening uptake; analysis of data from the surveys; analysis of data from the Hub once received.

## Supporting information

S1 File Feasibility study flow chart. (DOCX)

S1 Fig Study design. (JPG)

S2 Fig Data management plan. (JPG)

S1 Appendix Process for identifying mosques for participation. (DOCX)

S2 Appendix Baseline survey. (PDF)

S3 Appendix Post intervention survey. (PDF)

S4 Appendix 6 month follow up survey. (PDF)
