# Supplementary material for: Testing a faith-placed education intervention for bowel cancer screening in Muslim communities using a two-group non-randomised mixed-methods approach: Feasibility study protocol
Source: PLoS One. 2024 Mar 15;19(3):e0293339. doi: 10.1371/journal.pone.0293339 (PMC10942091; doi:10.1371/journal.pone.0293339)
Supplement: S1 File — (DOCX) [file pone.0293339.s001.docx]

**Write up and Publication**

**Receipt of Hub data**

Data will be supplied only using the unique identifier.

**Analyse Data**

Data from the surveys & hub and information from the interviews will be analysed.

**Assign Control and Intervention Groups to Mosques**

Pragmatic allocation

**Identify Mosques to Participate**

In Peterborough & Luton

**Request for Hub data**

Data request produced from the online survey system will only include those participants who have consented for their data to be requested from the Bowel Screening Hub. A unique Identifier will also be included. Requesting whether the participant has received a FIT KIT and/or returned a FIT KIT within the specified time frame of intervention sessions and survey completion.

**Information from Surveys entered onto the Online Survey System**

Paper – entered manually and quality checked

Electronically – link direct to the survey on the online survey system completed by participant or assisted during the session

Phone call – entered by the project team/peer support worker in real time

**6 Month Follow up Survey Completed**

Conducted via phone call. Includes consent to be contacted again and consent to collect data from the Hub – if previously stated no but consented to be contacted again or left hub data question blank

**6 Month Follow up Survey Completed**

Conducted via phone call. Includes consent to be contacted again and consent to collect data from the Hub – if previously stated no but consented to be contacted again or left hub data question blank

**Focus Group Interviews**

Participants & Health Professionals interviewed to feedback on the intervention sessions

**Post Intervention Survey Completed**

Immediately after intervention session. Includes consent to be contacted again and asked if wanting to participate in Focus Groups

**Intervention Session**

Conducted by Health professionals raising awareness of the Bowel cancer screening programme, encouraging participants to attend screening and not ignore their screening invitation letter, show how the FIT KIT is used

**Baseline Survey Completed**

Immediately before the intervention session. Includes Consent Form for data collection from survey, consent to be contacted again and consent to collect data from the Hub

**Control Site**

Control surveys organised with Faith Leaders. Intervention did not take place.

**Baseline Survey Completed**

Includes Consent Form for data collection from survey, consent to be contacted again and consent to collect data from the Hub

**Intervention Site**

Intervention sessions organised with Faith Leaders
