# Supplementary figures and images for: Testing a faith-placed education intervention for bowel cancer screening in Muslim communities using a two-group non-randomised mixed-methods approach: Feasibility study protocol
Source: PLoS One. 2024 Mar 15;19(3):e0293339. doi: 10.1371/journal.pone.0293339 (PMC10942091; doi:10.1371/journal.pone.0293339)

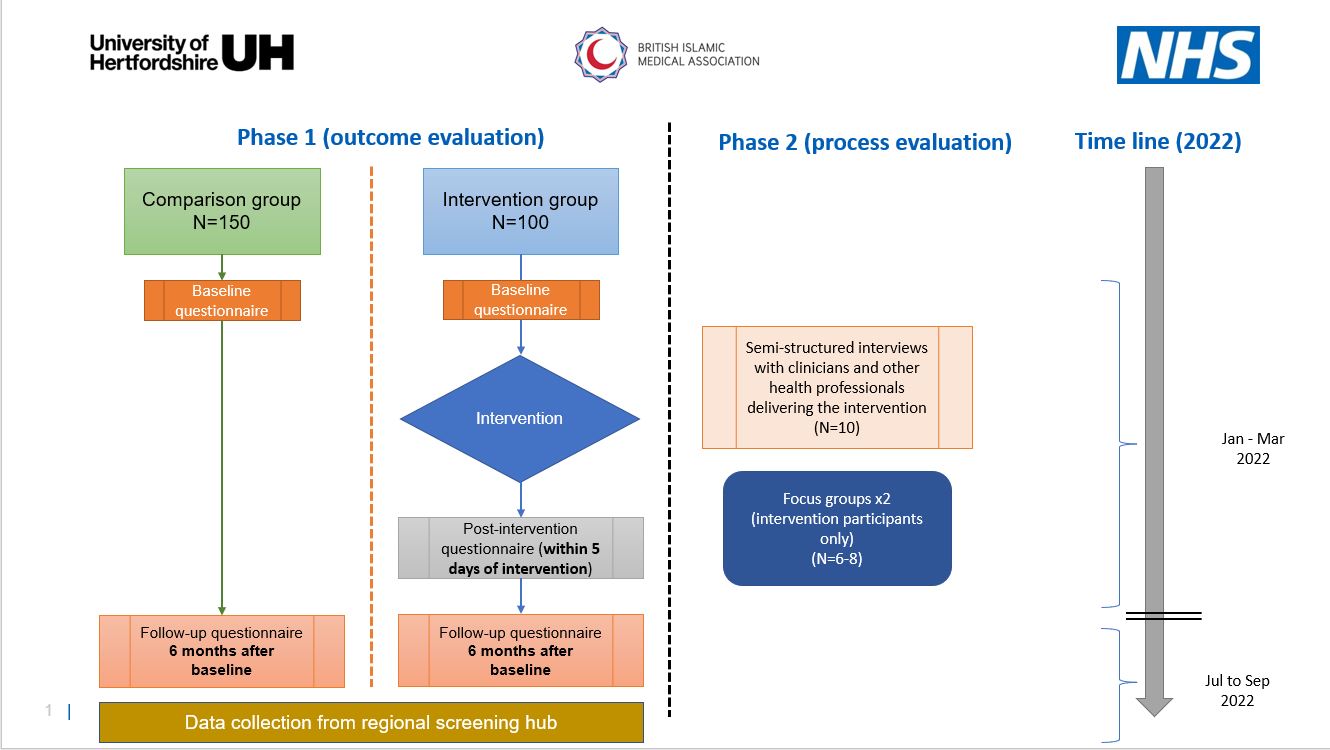

Supplement: S1 Fig — (JPG) [file pone.0293339.s002.JPG]

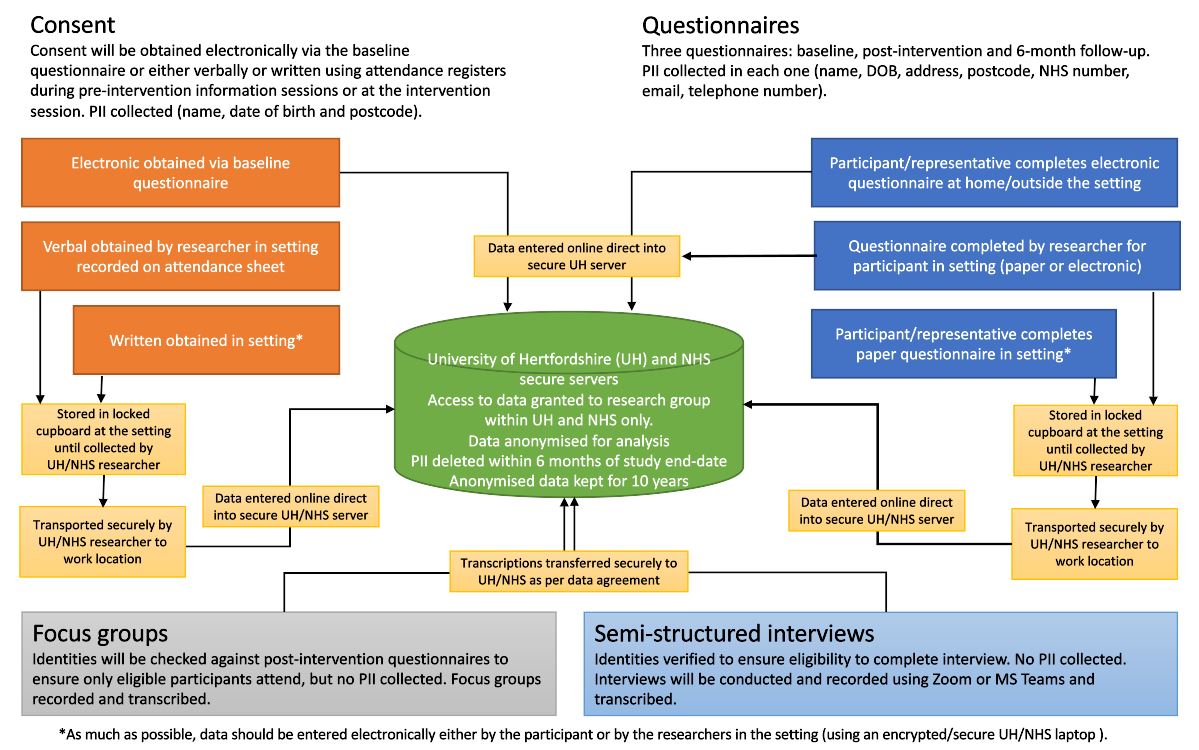

Supplement: S2 Fig — (JPG) [file pone.0293339.s003.JPG]
