# Supplementary material for: Testing a faith-placed education intervention for bowel cancer screening in Muslim communities using a two-group non-randomised mixed-methods approach: Feasibility study protocol
Source: PLoS One. 2024 Mar 15;19(3):e0293339. doi: 10.1371/journal.pone.0293339 (PMC10942091; doi:10.1371/journal.pone.0293339)
Supplement: S1 Appendix — (DOCX) [file pone.0293339.s004.docx]

# Testing a faith-placed education intervention for bowel cancer screening in Muslim communities: feasibility study

# Identifying mosques to approach for recruitment

## Introduction

Public Health England, alongside community stakeholders, identified Luton and Peterborough as areas that could benefit from the bowel cancer screening intervention, based on bowel cancer screening uptake, levels of deprivation, and ethnic diversity of the populations [1]. Specifically, low rates of screening uptake have been reported amongst UK Muslims [2-4] and both Luton and Peterborough have a large population of Muslim residents (around 50,000 in Luton, 25% of total, and 17,000 in Peterborough, 9% of total) [5].

The study is looking to recruit approximately 250 participants across both locations and will target mosques that are in catchment areas of GP practices with low bowel cancer screening uptake using mapping tools (150 in the intervention group and 100 in the comparison group). As well as mapping the mosques, it will also be important that the clinicians delivering the intervention are part of the same community so this will also factor into the identification of mosques to work with for this study.

## Method

To identify GP populations with low uptake of bowel cancer screening (people aged 60-74 years screened for bowel cancer within 6 months of invitation), uptake data within GP populations in Luton and Peterborough were obtained from the [Public Health England Fingertips platform](https://fingertips.phe.org.uk/profile/cancerservices/data#page/6/gid/1938132830/pat/166/par/E38000102/ati/7/are/E81632/iid/92601/age/280/sex/4/cat/-1/ctp/-1/yrr/1/cid/4/tbm/1/page-options/car-do-0) [6]. The latest data available were for 2019/20.

Performance indicators for % uptake of bowel cancer screening are:

- acceptable if >=52%
- achieved if >=60%

Initially, all those GP populations that reported below acceptable levels of uptake (<52%) in 2019/20 were defined as low uptake.

Once GPs with low uptake were identified, these were viewed on a map in [Shape Atlas.net](https://shapeatlas.net/). All the mosques in Luton and Peterborough were added to the map separately as they are not included in ShapeAtlas. A 1.5km circle* was drawn around the GP surgeries with uptake below 52% and the mosques within those circles were included; 1.5km represents approximately 15-20 minutes walking distance. All mosques within the 1.5km circle were counted and ranked in order of how many times they were included in the catchment of all the low uptake GPs.

Mosques that were within 1.5km of three of the GP practices with low uptake were identified as mosques to approach for our study.

*Actual catchment areas of each GP practice were unknown (these are defined differently by different practices and take into account a number of factors such as population density, health needs of the population, as well distance and other factors), so this was a pragmatic approach to estimate catchment areas of GP surgeries for the purpose of this exercise and are indicative only.

## Results

### Luton

In Luton, there are 26 practices with uptake of bowel cancer screening ranging between 35.4% and 70.8% in 2019/20. Nine practices reported <52% uptake [Table 1].

Table 1 Uptake of bowel cancer screening in GP practices in Luton: 2019/20

| **GP practice** | **Postcode** | **% uptake within 6 months invitation 2019/20** |
| --- | --- | --- |
| E81065 - Dr Sa Subramony'S Practice (Kingsway HC) | LU4 8BY | 35.40 |
| E81001 - Dr I Saleh's Practice (Wenlock) | LU2 0NN | 38.35 |
| Y02332 - Phoenix Primary Care (South) Ltd (Kingsway) | LU4 8BY | 42.24 |
| E81063 - Dr K Prasad's Practice (Conway MC) | LU4 8JD | 44.49 |
| E81028 - Dr R Khanchandani's Practice (Biscot Group) | LU3 1HA | 47.07 |
| Y02463 - The Town Centre Practice | LU1 2SE | 50.00 |
| E81631 - Malzeard Road Practice | LU3 1BD | 50.47 |
| E81612 - Drs Mirza Sukhani & Partners | LU4 9NN | 51.40 |
| E81073 - The Medici Medical Practice | LU1 3UA | 51.51 |
| E81048 - Bute House Medical Centre | LU1 1RW | 52.36 |
| E81016 - Lister House Surgery | LU4 8DG | 53.33 |
| E81032 - Lea Vale Medical Practice | LU1 1HH | 54.05 |
| E81633 - Neville Road Surgery | LU3 2JG | 55.74 |
| E81005 - Bell House Medical Centre | LU1 1BW | 56.17 |
| E81018 - Dr Jk Marsden's Practice | LU3 1RW | 56.31 |
| E81010 - Dr Whm Matta's Practice | LU4 9QZ | 57.22 |
| E81041 - Gardenia Practice | LU3 2NS | 58.10 |
| E81025 - The Oakley Surgery | LU4 9FJ | 60.06 |
| E81013 - Castle Medical Group Practice | LU1 3AG | 62.98 |
| E81632 - Barton Hills Medical Group | LU3 4AD | 63.18 |
| E81076 - Dr Dv Shah's Practice | LU4 0PF | 63.95 |
| E81040 - Sundon Medical Centre | LU3 3AH | 64.55 |
| E81026 - Larkside Practice | LU2 9SB | 66.08 |
| E81064 - Phoenix Primary Care (South) Ltd | LU3 4BG | 69.57 |
| E81617 - Dr Ps Bath's Practice | LU2 9AU | 70.19 |
| E81006 - Stopsley Village Practice | LU2 9AU | 70.75 |

Twelve mosques are situated within 1.5km of one of the nine GP practices, with seven situated in the catchment areas of three GP practices [Table 2].

Table 2. Mosques within 1.5km of low uptake GP practices in Luton

| **Mosque** | **Address** | **Postcode** | **Count within 1.5km of low uptake practices** |
| --- | --- | --- | --- |
| Madinah Mosque xP | 128-130 Oak Rd | LU4 8AD | 7 |
| Bury Park Mosque x B | 37C Upper George St | LU1 2RD | 6 |
| Al-Jalal Masjid | 314 Biscot Rd | LU3 1AZ | 5 |
| Masjid e Noor | 20 Cromwell Rd | LU3 1DN | 5 |
| Luton Central Mosque | 2-12 Westbourne Rd | LU4 8JD | 4 |
| Al-Hira Masjid & Centre | 1-7 Beechwood Rd | LU4 8RR | 3 |
| Baitul Abraar Jami Masjid | 364-370 Leagrave Rd | LU3 1RF | 3 |
| Farley Hill Jame Masjid | 31 The Cross Way | LU1 5LY | 2 |
| Hockwell Ring Masjid | 13-17 Barley Ln | LU4 9HT | 1 |
| Leagrave Hall Masjid | 145 High St | LU4 9LE | 1 |
| Masjid Bilal | 4 Sarum Rd | LU3 2RA | 1 |
| Yusuf Hull Masjid | Hill Rise | LU3 3EE | 1 |

### Peterborough

Uptake of bowel cancer screening was slightly better in Peterborough in 2019/20 and only two GP practices reported uptake less than 52%. In order to have a broad choice of mosques to approach, for Peterborough low uptake was defined as less than the achievable level of uptake of 60%.

Of 18 practices in Peterborough (note that some have several branches), uptake ranged from 44.3% to 72.0%. Two reported less than 52% and a further six reported below 60% [Table 3].

Table 3. Uptake of bowel cancer screening in GP practices in Peterborough: 2019/20

| **GP practice** | **Postcode** | **Uptake within 6 months invitation 2019/20** |
| --- | --- | --- |
| D81625 - Thistlemoor Medical Centre | PE1 3HP | 44.34 |
| D81631 - Central Medical Centre | PE1 3BF | 48.94 |
| D81065 - Nightingale Medical Centre | PE1 4FS | 54.74 |
| D81645 - The Grange Medical Centre | PE3 6HA | 55.41 |
| D81630 - Hampton Health | PE7 8DR | 55.56 |
| Y00486 - Botolph Bridge Community Health Centre | PE2 9QB | 58.39 |
| D81073 - Westwood Clinic | PE3 7JW | 59.41 |
| D81023 - Paston Health Centre | PE4 7DG | 59.63 |
| D81029 - Old Fletton Surgery | PE2 8AY | 63.59 |
| D81026 - Boroughbury Medical Centre | PE1 2EJ | 64.22 |
| D81629 - The Willow Tree Surgery | PE2 5RQ | 64.71 |
| D81022 - Octagon Medical Practice | PE4 5EG | 65.18 |
| D81046 - New Queen Street Surgery | PE7 1AT | 66.97 |
| D81618 - Ailsworth Medical Centre | PE5 7AF | 69.79 |
| D81615 - Thorpe Road | PE3 6AP | 70.50 |
| D81031 - Yaxley Group Practice | PE7 3JL | 71.83 |
| K83023 - Oundle | PE8 4JA | 71.95 |
| K83017 - Wansford | PE8 6PL | 71.99 |

Nine mosques are situated within 1.5km of one of the eight GP practices, with six situated in the catchment areas of three GP practices [Table 4].

Table 4. Mosques within 1.5km of low uptake GP practices in Peterborough

| Mosque | Address | Postcode | Count within 1.5km of low uptake practices |
| --- | --- | --- | --- |
| Masjid Ghousia x | 406 Gladstone St | PE1 2BY | 4 |
| Imam Ridha Center | 57 St Martins St | PE1 3BB | 3 |
| Masjid Darassalaam | 80-82 Alma Rd | PE1 3AW | 3 |
| Masjid Khadijah x | 311 Cromwell Rd | PE1 2HP | 3 |
| Qasimani Madrassa | 2 Bamber St | PE1 2HU | 3 |
| Tawheed Foundation | 1131 Bourges Blvd | PE1 2AX | 3 |
| Faizan E Madina Mosque x | 169-175 Gladstone St | PE1 2BN | 2 |
| Madina Madrassa and Spiritual Centre | 116 Midland Rd | PE3 6DD | 2 |
| Salah ad-Din Mosque | 60 Cromwell St | PE1 2EA | 2 |

Hussaini mosque

Ghousia and faizan are biggest

## Discussion

Thirteen mosques (seven in Luton and six in Peterborough) have been identified as potential study sites. Further discussions will be required within the research team to determine if there are clinicians in the community that are affiliated with these mosques and if the mosque and its local community will be willing to engage with the study.

Further assessment of the mosques will also be required to ascertain appropriateness of these sites in terms of their size and community reach and availability of rooms to undertake the intervention.

This was a pragmatic approach to identifying mosques In Luton and Peterborough close to GP practices whose uptake of bowel cancer screening in their population is low. These data will act as a starting point for recruitment of study sites. It is recognised that the study sites may ultimately be chosen based on those willing to engage in the study, potentially introducing selection bias.

## Next steps

- Research team to review this report in context of existing links with clinicians taking part in the study and to finalise list of mosques to approach.
- Research team to engage with chosen mosques and arrange informal sessions to talk through the study and what is required.

## References

1. Protocol to be added
2. Szczepura A, Johnson M, Orbell S, Gumber A, O’Sullivan I, Clay D, et al. Ethnicity: UK Colorectal Cancer Screening Pilot Final Report. The UK CRC Screening Pilot Evaluation (Ethnicity) Team. 2003 [cited 2021 Sep 14]; Available from: <http://wrap.warwick.ac.uk/133/1/WRAP_Szczepura_ethnicity-finalreport.pdf>
3. Szczepura A, Price C, Gumber A. Breast and bowel cancer screening uptake patterns over 15 years for UK south Asian ethnic minority populations, corrected for differences in socio-demographic characteristics. BMC Public Health [Internet]. 2008 Dec 2;8(1):346. Available from: <https://bmcpublichealth.biomedcentral.com/articles/10.1186/1471-2458-8-346>
4. Alexander F, Weller D. Evaluation of the UK Colorectal Cancer Screening Pilot [Internet]. University of Warwick institutional repository: http://go.warwick.ac.uk/wrap. 2003. Available from: <https://legacyscreening.phe.org.uk/policydb_download.php?doc=384>
5. [QS208EW (Religion) - Nomis - Official Labour Market Statistics (nomisweb.co.uk)](https://www.nomisweb.co.uk/census/2011/qs208ew)
6. [Public Health England Fingertips platform](https://fingertips.phe.org.uk/profile/cancerservices/data#page/6/gid/1938132830/pat/166/par/E38000102/ati/7/are/E81632/iid/92601/age/280/sex/4/cat/-1/ctp/-1/yrr/1/cid/4/tbm/1/page-options/car-do-0)
