# Supplementary material for: Testing a faith-placed education intervention for bowel cancer screening in Muslim communities using a two-group non-randomised mixed-methods approach: Feasibility study protocol
Source: PLoS One. 2024 Mar 15;19(3):e0293339. doi: 10.1371/journal.pone.0293339 (PMC10942091; doi:10.1371/journal.pone.0293339)
Supplement: S2 Appendix — (PDF) [file pone.0293339.s005.pdf]

Bowel cancer screening study – survey 1 (baseline)

About this research: information for participants

Welcome! Thank you for your interest in our work. Before you go any further, **please read the information for participants**, which accompanies this survey. This will explain what this research is about and what we are asking you to do.

You will then be asked to provide consent before taking part in this research.

The survey will take around 20-25 minutes to complete.

If you have any questions while you are completing this survey, please speak to the peer researcher at your mosque or contact the study team at: project.bima@nhs.net

Thank you for your valuable time in taking part in this study.

About you

1. **Are you completing this survey for yourself (as the participant) or on behalf of someone else (for the participant)?** (Please tick one option)

I am answering for myself	
I am answering for someone else	

2. **If you are answering for someone else, please state your name and your relationship to the participant.**

Privacy notice

The research team will respect the information you share with us. All information will be kept confidential and will not be available to anyone except the research team. All information will be anonymised. The information you provide will only be used for this study and other research or reports where individual participants cannot be identified, and for no other purpose. Individual participants will not be identified at any time.

Consent and eligibility

Before you consent to this research, **please make sure you have read the participant information sheet**. We also need to check you are eligible to take part. If you are completing this form for someone else, please ensure they have understood all the information in the participant information sheet before they provide consent.

The consent form is on the next page.

Participant consent and eligibility form

If you agree with all the following statements, please provide your consent at the end of this page. This lets the researchers know that you are happy to take part. If you would like to ask any questions before you agree to take part, please speak to the peer researcher at your mosque or contact: project.bima@nhs.net

I understand that:

- I can decide whether to take part in this study or not.
- I can stop taking part in the study at any time without giving a reason.
- I can ignore any questions I don't want to answer.
- Information about me collected as part of this study will be kept securely by the researchers.
- If any of the information I share with you gives you concerns about me or someone in my household, you will discuss this with me. If I agree, you may share this information with someone that can help.

I agree that:

- Information that does not identify me can be kept for use by other researchers.
- Information I give can be used in reports and other materials (including teaching materials) if I cannot be identified.
- The research team can contact me and ask me to complete another survey after the session (intervention group only) and again in six months (intervention and comparison group).
- The research team can contact me to ask me if I want to take part in a focus group interview at a later date (intervention group only).
- I have had time to ask questions about this study and I am happy to take part.

I confirm that:

- I am 56 years of age or older. (If you are completing the form for someone else, you confirm the person you are completing it for is 56 years of age or older).

3. **Please indicate whether you/the person you represent consent/s to take part in this research** (please tick correct box).

I agree to all the statements above and I consent to take part in this research.	
I do not give my consent to take part.	
I have more questions about this research and not ready to consent to take part. Please contact research team at: project.bima@nhs.net	

If you have consented to take part, please turn to page 4 to start the survey.

Participant details

Please remember if you are completing this for someone else to enter their details not yours. Thank you.

**4. What is your first (or given) name?**	
**5. What is your surname (family name)?**	

**6. Home address:**	
**7. Postcode**	

**8. What is your date of birth?** (dd/mm/yyyy)	
**9. What is your age (in years)?**	

10. **Are you registered with a GP?** (Please tick one)

☐ Yes ☐ No ☐ Prefer not to say

**10. a) Who is your GP?**<br>(Please give name of practice)	
**10. b) Address of GP practice**<br>(Please include postcode if known)	

11. **If you know your NHS number, please enter it here** (it should be 10 digits).

Equality

It is important that people from all backgrounds can access the health services they need. Everyone should receive fair treatment whatever their personal situation.

We need to know what these situations and backgrounds are so that healthcare services can be informed on the best ways to provide care for different groups of people. It is important no one is disadvantaged because they cannot access important health information.

The next few questions help us to do this for bowel screening.

The questions may seem personal but all your answers will be anonymised so they cannot be traced back to you. Please be as honest as you can. All questions have the option: 'Prefer not to say' if you do not feel comfortable with some of the questions.

Remember if you are responding on behalf of the participant, please answer for them.

12. What is your gender? (Please tick one)

☐ Man ☐ Woman ☐ Other (please describe) ☐ Prefer not to say

12. a) If you selected Other, please specify:

13. What is your ethnic background? (Please tick one)

Asian / Asian British - Indian		Asian / Asian British - Pakistani	
Asian / Asian British - Bangladeshi		Asian / Asian British - Chinese	
Asian / Asian British - any other Asian background (please describe below)		Arab	
Black / African / Caribbean / Black British - African		Black / African / Caribbean / Black British - Caribbean	
Black / African / Caribbean / Black British - other Black background (please describe)			
White - English / Welsh / Scottish / Northern Irish / British		White - any other White background (please describe below)	
Mixed - White and Black Caribbean		Mixed - White and Black African	
Mixed - White and Asian (please describe below)		Mixed - any other mixed / multiple ethnic background (please describe below)	
Prefer not to say			
Other (please describe)		**13.a)**	

Please tell us which languages you speak, and which is your preferred language to speak.

Language	**14. I can speak** (tick all that apply)	**15. I prefer to speak** (tick one only)
English		
Urdu		
Bengali/Sylheti		
Arabic		
Gujarati		
Punjabi		
Hindi		
Turkish		
Somali		
Kurdish		
Pashto		
Farsi		
Prefer not to say		
Other (please describe)	**14.a)**	**15. a)**

16. **How long have you been living in the UK?** (Please tick one)

☐ From birth ☐ Less than 5 years ☐ 5-10 years ☐ Over 10 years

☐ I don't know/can't remember ☐ Prefer not to say

17. **Are you** (please tick one)

☐ Single (never married) ☐ Married or living with a partner

☐ Divorced or separated ☐ Widowed

☐ Prefer not to say

☐ Other (please describe): **17.a)** ………………………………………………………………

18. Do any of the following people live in your household with you? (Please tick all the boxes that apply)

Wife / husband / partner		Child(ren)	
Parent(s)		Childcare/nanny	
Carer		Friend(s)	
I live alone		Prefer not to say	
Other (please describe)		**18.a)**	

19. Please tick the box which best describes where you live. (Tick one box only)

I own my own home (including if with a mortgage)		I rent from a private landlord	
I rent from a housing association or council		I live in temporary housing	
Prefer not to say			
Other (please describe)		**19. a)**	

20. How would you describe your occupation? (Tick one box only)

Work full time		Work part time	
Unemployed		Studying full time	
Retired		Homemaker	
Prefer not to say			
Other (please describe)		**20.a)**	

21. What is your highest level of formal educational qualification? (Tick one box only)

☐ Postgraduate degree (e.g. PgDip, MSc, MA, MBA, PhD)

☐ Undergraduate degree (e.g. BSc, BA, BEng)

☐ Other type of higher education (e.g. HND, HNC, nursing qualifications)

☐ A Levels/NVQ3

☐ Further education (e.g. NVQ level 2, City and Guilds, BTec diploma)

☐ GCSEs/O levels/CSEs

☐ No formal qualifications

☐ Prefer not to say

☐ Other (please describe) **21. a)** ……………………………………………………………...

Bowel cancer screening

Bowel cancer screening involves two types of test or examination.

- Testing of a poo sample using a kit. Your kit may have been:
    a. a FIT (or Faecal Immunochemical Test) - you take **one** small sample of poo at home, which you then send off by post to be tested, or
    b. a FOBT (or Faecal Occult Blood Test) - you take **2 samples of poo on three separate days** at home, which you then send off by post to be tested.
- You may have been into hospital for a bowel scope. This is when a tube containing a small camera is inserted into the bowel through your back passage (rectum).

Physical examination of the back passage by your GP is not the same as bowel cancer screening.

22. Have you ever completed a bowel cancer screening test?

☐ Yes ☐ No ☐ I don't know ☐ Prefer not to say

If you ticked <u>Yes</u> for question 22, please answer questions 23 and 24, then move straight to question 27 on page 10.

If you ticked <u>No</u>, please answer questions 25 and 26 on page 9.

If you ticked <u>I don't know</u> or <u>Prefer not to say</u> please go to question 27 on page 10.

I have completed a bowel screening test

23. What type of screening test/s did you have? (Tick all that apply)

☐ Faecal Immunochemical Test (FIT)

☐ Faecal Occult Blood Test (FOBT)

☐ Bowel scope

☐ I don't know/can't remember ☐ Prefer not to say

24. When did your bowel screening take place? (If you don't know the exact date, please estimate as best you can.) **Please give details of all bowel screening tests you have completed.**

I have not completed a bowel screening test

25. **Please give a reason why you have not completed a bowel screening test.**
(Please tick one)

☐ I haven't been invited to complete a test

☐ I was invited to complete a test, but I declined

☐ I don't know ☐ Prefer not to say

26. If you answered that you declined screening in question 25, why was this?
(Please tick all that apply).

☐ I don't think I am at risk of bowel cancer

☐ I didn't think it was important

☐ It seemed too difficult

☐ I didn't have time

☐ I didn't understand what was involved in the screening

☐ I didn't understand what would happen if the test was positive

☐ I was scared in case it was positive

☐ The thought of collecting my own poo is disgusting

☐ Prefer not to say

☐ Not applicable

☐ Other reason (please describe):

**26. a)**

Please continue to question 27 on the next page.

Bowel cancer screening - knowledge and attitudes

27. Have you ever been told by a doctor that you have or may have bowel cancer?

☐ Yes ☐ No ☐ Prefer not to say

28. Are you currently having treatment for bowel cancer?

☐ Yes ☐ No ☐ Prefer not to say

29. Has anyone close to you (e.g. parent, sibling, partner, friend, other) currently or in the past been told by a doctor that they have bowel cancer?

☐ Yes ☐ No ☐ Prefer not to say ☐ I don't know

For questions 30 to 32, please tell us if you agree or disagree with the following three statements. (Please circle the option that applies to you)

30. I understand the reasons why people are invited to take part in bowel cancer screening.

Completely agree	Mostly agree	Undecided/ not sure	Mostly disagree	Completely disagree

31. I think I will participate in bowel screening when I am invited.

Completely agree	Mostly agree	Undecided/ not sure	Mostly disagree	Completely disagree

32. I think I will ask for a screening kit if I am eligible to receive one.

Completely agree	Mostly agree	Undecided/ not sure	Mostly disagree	Completely disagree

33. If you don't think you will ask for a screening kit or participate in bowel screening, or you're not sure, please give your reasons (tick all that apply).

I don't think I am at risk of bowel cancer		I don't understand what is involved in the screening	
I don't think it is important		I don't understand what will happen if the test is positive	
It seems too difficult		I am scared in case the test is positive	
I don't have time		The thought of collecting my own poo is disgusting	
Prefer not to say		Not applicable	
Other reason (please describe)		**33.a)**	

Follow up

We would like to follow up (check) whether you took part in screening.

If you agree we will contact the screening service to find out:

a. if you asked for a screening kit
b. if you did the test and returned the screening kit.

We will not see your medical records or what the results are from your screening test.

34. Can we follow up regarding whether or not you took part in screening?

☐ Yes ☐ No

Contact details

We ask for an email address and telephone number in case we need more information on the answers you have provided. We will also use these details to contact you regarding further surveys as part of this study only.

35. **Are you happy for us to contact you again?** ☐ Yes ☐ No

**36. Email address**	
**37. Telephone number**	

If you are representing the participant and completing the survey on their behalf, please enter your contact details here. If you are the participant, please enter your own contact details in this section.

Thank you for your time – you have now reached the end of the survey.

What happens now?

Your survey will be passed to the research team, who will enter the data into a database, where it will be stored securely until all the data are analysed.

If you have been allocated to the intervention group, you will receive further information about this shortly.

If you have been allocated to the comparison group, you will be invited to complete a follow up survey in six months' time.

We would like to thank you again for helping us with this important research. If you have any questions about the study later on, you can contact the research team at: project.bima@nhs.net
