# Supplementary material for: Testing a faith-placed education intervention for bowel cancer screening in Muslim communities using a two-group non-randomised mixed-methods approach: Feasibility study protocol
Source: PLoS One. 2024 Mar 15;19(3):e0293339. doi: 10.1371/journal.pone.0293339 (PMC10942091; doi:10.1371/journal.pone.0293339)
Supplement: S3 Appendix — (PDF) [file pone.0293339.s006.pdf]

Bowel cancer screening study – survey 2 (post education session)

About this research: information for participants

Welcome! You have been invited to complete this second survey because you have (or the person you are representing has) attended or viewed an education session on bowel cancer screening. We hope it was helpful for you.

Before you go any further, please feel free to read the information for participants again if you need to.

The survey will take around 15-20 minutes to complete.

If you have any questions while you are completing this survey, please speak to the peer researcher at your mosque or contact the study team at: project.bima@nhs.net

Thank you for your valuable time in taking part in this study.

Privacy notice

The research team will respect the information you share with us. All information will be kept confidential and will not be available to anyone except the research team. All information will be anonymised. The information you provide will only be used for this study and other research or reports where individual participants cannot be identified, and for no other purpose. Individual participants will not be identified at any time.

About you

1. **Are you completing this survey for yourself (as the participant) or on behalf of someone else (for the participant)?** (Please tick one option)

I am answering for myself	
I am answering for someone else	

2. **If you are answering for someone else, please state your name and your relationship to the participant.**

Eligibility

3. **Have you (or the person you are representing) attended or watched a bowel cancer screening education session?**

☐ Yes ☐ No

If you have not attended an education session on bowel cancer screening, please come back to this survey once you have attended the session. If you think you should have been invited to attend a session and you haven't received an invite, please contact the research team at: project.bima@nhs.net

Participant details

We are asking for this information so we can match your survey with the first survey you completed. Please remember if you are completing this for someone else to enter their details not yours. Thank you.

**4. What is your first (or given) name?**	
**5. What is your surname (family name)?**	

**6. Home address:**	
**7. Postcode**	

**8. What is your date of birth?** (dd/mm/yyyy)	

9. **If you know your NHS number, please enter it here** (it should be 10 digits).

About the bowel cancer education session

10. **When did you attend or view the education session? Please give dates** (dd/mm/yyyy)

11. **Where did you receive the education session?** (Please tick one option)

I attended a session in person	
I attended a live session online	
I watched a recording online	
Someone else attended a session in person for me	
Someone else attended a live online session for me	
Someone else viewed a recording for me	

If you attended a session in person, please continue with questions 12 and 13.

If you attended a session online or watched a recording, please skip questions 12 and 13 and go straight to question 14.

In person session

12. **Where was the education session held?** (Please tick one)

Luton – mosque A	
Luton – mosque B	
Luton – mosque C	
Peterborough – mosque A	
Peterborough – mosque B	
Peterborough – mosque C	

13. **Please tell us if you agree or disagree with the following statement: The venue was in a convenient location for me.** (Please circle or tick one option)

Completely agree	Mostly agree	Undecided/ not sure	Mostly disagree	Completely disagree

Please now skip question 14 and continue to question 15; then complete the rest of the survey.

Live stream or pre-recorded session

14. Please tell us whether you agree or disagree with the following statement: The education session was easy for me to access. (Please circle or tick one option)

Completely agree	Mostly agree	Undecided/ not sure	Mostly disagree	Completely disagree

Please continue to question 15 and complete the rest of the survey.

About the bowel cancer education session

15. What language or languages was the education session delivered in? (Tick all that apply)

English		
Urdu		
Bengali/Sylheti		
Arabic		
Gujarati		
Punjabi		
Hindi		
Turkish		
Somali		
Kurdish		
Pashto		
Farsi		
I don't know		
Other (please describe)		**15. a)**

16. Would you have preferred another language?

☐ Yes ☐ No ☐ I don't know ☐ Prefer not to say

16.a) If you selected yes to question 16, which language/s would you have preferred?

For questions 17 to 20, please tell us if you agree or disagree with the following statements. (Please circle or tick the option that applies to you)

17. I found the education session useful.

Completely agree	Mostly agree	Undecided/ not sure	Mostly disagree	Completely disagree

18. The education session was tailored to my needs.

Completely agree	Mostly agree	Undecided/ not sure	Mostly disagree	Completely disagree

19. It was helpful to have a doctor or nurse who is part of my community talk to me about bowel cancer screening.

Completely agree	Mostly agree	Undecided/ not sure	Mostly disagree	Completely disagree

20. I felt comfortable enough to ask any questions during the session.

Completely agree	Mostly agree	Undecided/ not sure	Mostly disagree	Completely disagree	Not applicable

21. Would you like to add any other comments about how you found the education session?

Bowel cancer screening - knowledge and attitudes

For questions 22 to 24, please tell us if you agree or disagree with the following statements. (Please circle or tick the option that applies to you)

22. I understand the reasons why people are invited to take part in bowel cancer screening.

Completely agree	Mostly agree	Undecided/ not sure	Mostly disagree	Completely disagree

23. I think I will participate in bowel screening when I am invited.

Completely agree	Mostly agree	Undecided/ not sure	Mostly disagree	Completely disagree

24. I think I will ask for a screening kit if I am eligible to receive one.

Completely agree	Mostly agree	Undecided/ not sure	Mostly disagree	Completely disagree

25. If you don't think you will ask for a screening kit or participate in bowel screening, or you're not sure, please give your reasons (tick all that apply).

I don't think I am at risk of bowel cancer		I don't understand what is involved in the screening	
I don't think it is important		I don't understand what will happen if the test is positive	
It seems too difficult		I am scared in case the test is positive	
I don't have time		The thought of collecting my own poo is disgusting	
Prefer not to say		Not applicable	
Other reason (please describe)		**25.a)**	

Follow up

We would now like to invite you to take part in a focus group (a small group of 6 to 8 people). This will be a chance for you to tell us what you think about the education session, and what some of the things are that might help you take part in bowel screening, and what may make it difficult for you to take part.

Any information you give in the focus group will be kept confidential. Please see the participant information sheet for more information about this.

Please provide consent to take part in a focus group over the page.

26. Do you agree to be contacted to take part in a focus group as part of this research?

☐ Yes ☐ No ☐ Maybe

If you selected Yes or Maybe, please provide your contact details below.

Contact details

We ask for an email address and telephone number in case we need more information on the answers you have provided. We will also use these details to contact you regarding further surveys or focus groups as part of this study only.

27. **Are you happy for us to contact you again?** ☐ Yes ☐ No

**28. Email address**	
**29. Telephone number**	

If you are representing the participant and completing the survey on their behalf, please enter your contact details here. If you are the participant, please enter your own contact details in this section.

You have now come to the end of the survey.

What happens now?

Thank you for completing this survey. The information you have provided will help us find out the best ways of providing information about bowel cancer screening to different groups of the UK population. We hope that improved knowledge about screening will increase the uptake, which means more people can be offered treatment early. Early treatment has a greater chance of success and can save lives.

If you have consented to take part in one of our focus groups, someone from the research team will be in touch with you in the next few weeks. You will also be invited to complete the final follow-up survey in six months' time. If you have any questions about this study in the meantime, you can contact the research team at: project.bima@nhs.net

Thank you again for your time, it is very much appreciated.
