# Supplementary material for: Testing a faith-placed education intervention for bowel cancer screening in Muslim communities using a two-group non-randomised mixed-methods approach: Feasibility study protocol
Source: PLoS One. 2024 Mar 15;19(3):e0293339. doi: 10.1371/journal.pone.0293339 (PMC10942091; doi:10.1371/journal.pone.0293339)
Supplement: S4 Appendix — (PDF) [file pone.0293339.s007.pdf]

Bowel cancer screening study – 6-months follow-up survey

About this research: information for participants

Welcome! You have been invited to complete this survey because you have (or the person you are representing has) consented to be part of our study on bowel cancer screening.

If you need to, please feel free to read the participant information again before you complete the survey.

This is the final survey and should only take 10-15 minutes to complete.

You may notice that some of the questions are the same as those in the first survey - this is so we can see if there have been any changes over time. Please answer as honestly as you can.

If you have any questions while you are completing this survey, please speak to the peer researcher at your mosque or contact the study team at: project.bima@nhs.net

Thank you for your valuable time in taking part in this study.

Privacy notice

The research team will respect the information you share with us. All information will be kept confidential and will not be available to anyone except the research team. All information will be anonymised. The information you provide will only be used for this study and other research or reports where individual participants cannot be identified, and for no other purpose. Individual participants will not be identified at any time.

About you

1. **Are you completing this survey for yourself (as the participant) or on behalf of someone else (for the participant)?** (Please tick one option)

I am answering for myself	
I am answering for someone else	

2. **If you are answering for someone else, please state your name and your relationship to the participant.**

Participant details

We are asking for this information so we can match your survey with your previous survey/s. Please remember if you are completing this for someone else to enter their details not yours. Thank you.

**3. What is your first (or given) name?**	
**4. What is your surname (family name)?**	

**5. Home address:**	
**6. Postcode**	

**7. What is your date of birth?** (dd/mm/yyyy)	

8. **If you know your NHS number, please enter it here** (it should be 10 digits).

Bowel cancer screening

Bowel cancer screening in England currently involves a FIT (or Faecal Immunochemical Test) - you take **one** small sample of poo at home, which you then send off by post to be tested.

Physical examination of the back passage by your GP is not the same as bowel cancer screening.

9. Have you ever completed a bowel cancer screening test?

☐ Yes ☐ No ☐ I don't know ☐ Prefer not to say

If you ticked Yes for question 9, please answer question 10 on page 4 then move on to question 13 on page 5.

If you ticked No, please skip question 10 and move to questions 11 and 12 on page 4.

If you ticked I don't know or Prefer not to say please go straight to question 13 on page 5.

I have completed a bowel screening test

10. When did your bowel screening take place? (If you don't know the exact date, please estimate as best you can.) **Please give details/dates (dd/mm/yyyy) of all bowel screening tests you have completed.**

I have not completed a bowel screening test

11. **Please give a reason why you have not completed a bowel screening test.** (Please tick one)

I haven't been invited to complete a test		I was invited to complete a test, but I declined	
I don't know		Prefer not to say	

12. If you answered that you declined screening in question 11, why was this? (Please all that apply).

I don't think I am at risk of bowel cancer		I didn't understand what was involved in the screening	
I didn't think it is important		I didn't understand what would happen if the test was positive	
It seemed too difficult		I was scared in case it was positive	
I didn't have time		The thought of collecting my own poo is disgusting	
Prefer not to say		Not applicable	
Other reason (please describe)		**12.a)**	

Please now continue with the rest of the questions on the following pages.

Bowel cancer screening - knowledge and attitudes

13. Have you ever been told by a doctor that you have or may have bowel cancer?

☐ Yes ☐ No ☐ Prefer not to say

14. Are you currently having treatment for bowel cancer?

☐ Yes ☐ No ☐ Prefer not to say

15. Has anyone close to you (e.g. parent, sibling, partner, friend, other) currently or in the past been told by a doctor that they have bowel cancer?

☐ Yes ☐ No ☐ Prefer not to say ☐ I don't know

For questions 16 to 18, please tell us if you agree or disagree with the following statements. (Please circle or tick the option that applies to you)

16. I understand the reasons why people are invited to take part in bowel cancer screening.

Completely agree	Mostly agree	Undecided/ not sure	Mostly disagree	Completely disagree

17. I think I will participate in bowel screening when I am invited.

Completely agree	Mostly agree	Undecided/ not sure	Mostly disagree	Completely disagree

18. I think I will ask for a screening kit if I am eligible to receive one.

Completely agree	Mostly agree	Undecided/ not sure	Mostly disagree	Completely disagree

19. If you don't think you will ask for a screening kit or participate in bowel screening, or you're not sure, please give your reasons (tick all that apply).

I don't think I am at risk of bowel cancer		I don't understand what is involved in the screening	
I don't think it is important		I don't understand what will happen if the test is positive	
It seems too difficult		I am scared in case the test is positive	
I don't have time		The thought of collecting my own poo is disgusting	
Prefer not to say		Not applicable	
Other reason (please describe)		**19.a)**	

Contact details

We ask for an email address and telephone number in case we need more information on the answers you have provided. We will also contact you to invite you to an education session if you have not already attended one and to share the results of this study. We will not use your contact details for any other reason.

20. **Are you happy for us to contact you again?** ☐ Yes ☐ No

**21. Email address**	
**22. Telephone number**	

If you are representing the participant and completing the survey on their behalf, please enter your contact details here. If you are the participant, please enter your own contact details in this section.

You have now come to the end of this survey.

What happens now?

Thank you for completing this survey. The information you have provided will help us find out the best ways of providing information about bowel cancer screening to different groups of the UK population. We hope that improved knowledge about screening will increase the uptake, which means more people can be offered treatment early. Early treatment has a greater chance of success and can save lives.

If you have consented to receive further information from us on attending an information session or receiving the results of the study, we will be in touch with you in due course.

If you have any questions about this study in the meantime, you can contact the research team at: project.bima@nhs.net

Thank you again for your time, it is very much appreciated.
